# Transcriptomic Landscape of Medicinal *Dendrobium* Reveals Genes Associated With the Biosynthesis of Bioactive Components

**DOI:** 10.3389/fpls.2020.00391

**Published:** 2020-04-28

**Authors:** Zhicai Wang, Meili Zhao, Hongqiu Cui, Jian Li, Meina Wang

**Affiliations:** ^1^Key Laboratory of National Forestry and Grassland Administration for Orchid Conservation and Utilization, Shenzhen, China; ^2^Shenzhen Key Laboratory for Orchid Conservation and Utilization, The National Orchid Conservation Center of China and The Orchid Conservation & Research Center of Shenzhen, Shenzhen, China; ^3^Key Laboratory of Medical Reprogramming Technology, Shenzhen Second People’s Hospital, The First Affiliated Hospital of Shenzhen University, Shenzhen, China

**Keywords:** *Dendrobium*, specialized metabolites, medicinal components, biosynthesis, transcriptome

## Abstract

Many plants of *Dendrobium* genus are precious traditional herbs with high commercial value and excellent medicinal effects. They are perennial aerophytes or epiphytes of terrestrial orchids growing on cliffs and tree trunks covered with mosses in forests throughout the tropical and subtropical Asia and eastern Australia. The stem contains a variety of bioactive components, including polysaccharides and alkaloids, with strong antioxidant, neuroprotective, and immunomodulatory effects. Great attention has been drawn to the *Dendrobium* genus regarding its medicinal effectiveness, and the related researches have been accumulating rapidly in recent years. The bioactive components are mainly the intermediates or final products produced in specialized metabolite biosynthesis. Thus far, the activity, molecular structure, and composition of major medicinal ingredients have been partially elucidated, and the sequencing of several transcriptomes has been starting to shed new light on the biosynthesis regulation mechanism. This paper reviewed the advances of researches concerning the biosynthetic pathways of medicinal specialized metabolites from *Dendrobium*, especially the large number of related genes, with the hope of further promoting the development and utilization of those components and correspondingly protecting the *Dendrobium* resources in more effective ways.

## Introduction

*Dendrobium*, one of the largest genera in the *Orchidaceae* family with more than 1500 species worldwide, is mainly distributed throughout India, southern Asia, Japan, Australia, and some Pacific islands (Wang et al., 2009; Pridgeon et al., 2014). Several *Dendrobium* species including *D. nobile*, *D. officinale* (*D. catenatum*), *D. huoshanense*, and *D. chrysanthum*, are highly prized folk medicines (Table 1) in many Asian countries for hundreds of years with special pharmacological effects on inflammation, gastritis, diabetes, cancer, and aging (Ng et al., 2012; Song et al., 2012).

**TABLE 1:** The effective components and their bioactivities in medicinal *Dendrobium.*

Bioactive constituents	Species names	Bioactivity	References
Alkaloids	*D. nobile*	Neuroprotective activity	Li et al., 2011
Bisbenzyls	*D. nobile*	Antifungal activities	Zhou et al., 2016
Dendroflorin	*D. nobile*	Antisenescence	Jin et al., 2008
Flavonoids	*D. officinale*	Antioxidant, antitoxicity	Prochazkova et al., 2011; Wang et al., 2017
Glucosyloxycinnamic acid derivatives	*D. aurantiacum*	Antioxidant	Yang et al., 2004
Lectin	*D. findleyanum, D. officinale*	Hemagglutinating, antifungal	Sattayasai et al., 2009
Moscatilin	*D. loddigesii*	Suppresses tumor angiogenesis and growth	Tsai et al., 2010
Polysaccharides	*D. huoshanense, D. officinale*	Immunomodulatory, hepatoprotective, and antioxidant activities	Liu et al., 2011; Wu et al., 2011; Pan et al., 2012
Phenanthrenes	*D. loddigesii*	Antioxidant	Ito et al., 2010
Trigonopol A	*D. trigonopus*	Inhibits platelet aggregation	Hu et al., 2008

The major active ingredients include polysaccharides (Luo et al., 2009), alkaloids (Wang Q. et al., 2010), bibenzyls (Yang et al., 2006), flavonoids (Lei et al., 2018), amino acids, and several trace mineral elements (Guo et al., 2013). Due to over-exploitation and deterioration of natural habitats, most of the wild *Dendrobium* species have been increasingly endangered.

**FIGURE 1:**
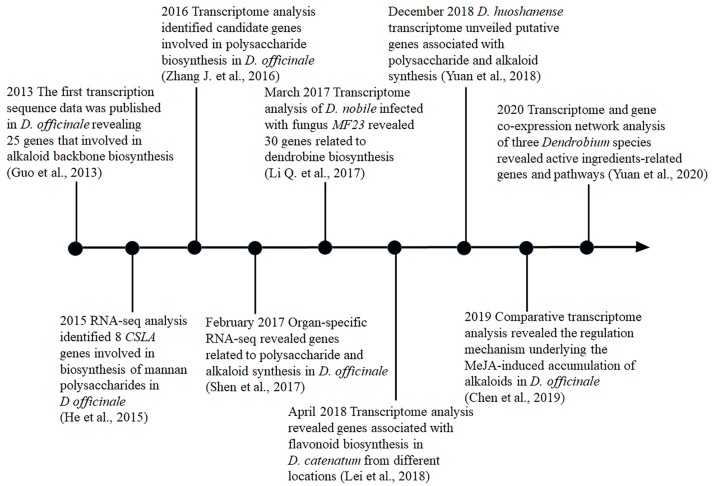
Timeline of transcriptome studies probing the biosynthesis pathways for active ingredients in medicinal *Dendrobium*. Notable studies were included and the key references for each study were given.

Transcriptomic analysis is a powerful tool for exploring specialized metabolite biosynthetic genes and their expression patterns, which can be used to determine the synthesis and metabolic pathway. Recently, a large number of putative genes involved in the biosynthesis of polysaccharides (Shen et al., 2017), alkaloids (Li Q. et al., 2017), and flavonoids (Lei et al., 2018) have been identified in *Dendrobium* through transcriptome sequencing (Table 2 and Figure 1). For instance, in *D. officinale*, the first transcription sequencing data that revealed the genes associated with alkaloid biosynthesis were published in 2013 (Guo et al., 2013). In 2016, a transcriptome study focusing on the regulatory maps in response to cold acclimation, polysaccharide synthesis, and gene expression profiling of the protocorm has been conducted (Zhang J. et al., 2016). In that same year, the genome of *D. catenatum* was sequenced and the polysaccharide synthetic pathway was analyzed (Zhang G.Q. et al., 2016). Later, in 2017 (Shen et al., 2017), unigenes associated with fructose and mannose metabolism and the putative alkaloid biosynthetic pathway were identified, while in *D. nobile*, transcriptome analysis was carried out to reveal genes related to the biosynthesis of dendrobine through the mevalonate (MVA) pathway (Li Q. et al., 2017). Furthermore, genes in the polysaccharide synthetic pathway, including *cellulose synthase-like A 6* (*DoCSLA6*), *UDP galacturonate 4-epimerase* (*DoUGE*), *UDP-glucose pyrophosphorylase* (*DoUGP*), and *GDP-mannose pyrophosphorylase 1* (*DoGMP1*) have been cloned and functionally characterized in *D. officinale* (Fan et al., 2016). The purpose of this review is to summarize the advances in transcriptome-related studies, with an emphasis on functional characterization of regulatory genes related to some of the major active ingredient biosynthesis in recent years and to provide useful insights into the further dissection of biosynthesis regulation mechanism in *Dendrobium*.

**TABLE 2:** Transcriptome sequencing of medicinal *Dendrobium* revealing genes related to specialized metabolites production.

*Dendrobium* species	Sequencing time	Sequencing country	Sequencing platforms	References
*D. officinale*	2013	China	Roche 454 GS FLX Titanium platform	Guo et al., 2013
*D. officinale*	2015	China	Illumina HiSeq 2000	He et al., 2015
*D. officinale*	2016	China	Illumina HiSeq 2000	Zhang J. et al., 2016
*D. officinale*	2016	China	Illumina HiSeq 2000	An et al., 2016
*D. officinale*	2017	China	Illumina HiSeq 2500	Shen et al., 2017
*D. nobile*	2017	China	Illumina HiSeq 4000	Li Q. et al., 2017
*D. officinale*	2017	China	Illumina HiSeq 1500	He et al., 2017b
*D. huoshanense*	2018	China	Illumina HiSeq 2500	Yuan et al., 2018
*D. catenatum*	2018	China	Illumina HiSeq 4000	Lei et al., 2018
*D. officinale*	2019	China	Illumina HiSeq 4000	Chen et al., 2019
*D. huoshanense, D. officinale*, and *D. moniliforme*	2020	China	Not available	Yuan et al., 2020

## *Dendrobium* Alkaloid Biosynthesis

### *Dendrobium* Alkaloid

The major medicinal constituents of *Dendrobium* include alkaloids, flavonoids, polysaccharides, polyphenols, etc. (Lu et al., 2014). Among these compounds, alkaloids are the most important medicinal components and the first category extracted and characterized from *Dendrobium* plants. Thus far, five types of structurally confirmed alkaloids including sesquiterpene alkaloids, imidazole alkaloids, phthalide alkaloids, pyrrolidine alkaloids, and indolizidine alkaloids have been clarified (Ng et al., 2012). Despite the complex composition of alkaloids, sesquiterpene alkaloid dendrobine has been regarded as the quality standard for many *Dendrobium* plants (Li R. et al., 2017). Evidences from modern pharmacology have demonstrated that *Dendrobium* alkaloids have remarkable antihypertensive, anticancer, antipyretic, eye-benefiting, neuroprotective, and immune regulatory effects in preclinical studies (Wang J.H. et al., 2010; Li et al., 2011). Currently, alkaloids are primarily obtained through extraction and chemical synthesis (Kreis and Carreira, 2012). However, neither of these approaches is efficient enough because of extremely low accumulation levels in the *Dendrobium* plants and technical problems in total synthesis. Due to the high market demand, overexploitation, and deterioration of natural habitats, wild *Dendrobium* resources have been increasingly depleted. Therefore, the biotechnology-based strategy is promising for stably producing large quantities of alkaloids to meet the market demand and protect the wild resources.

### *Dendrobium* Alkaloid Biosynthesis

**FIGURE 2:**
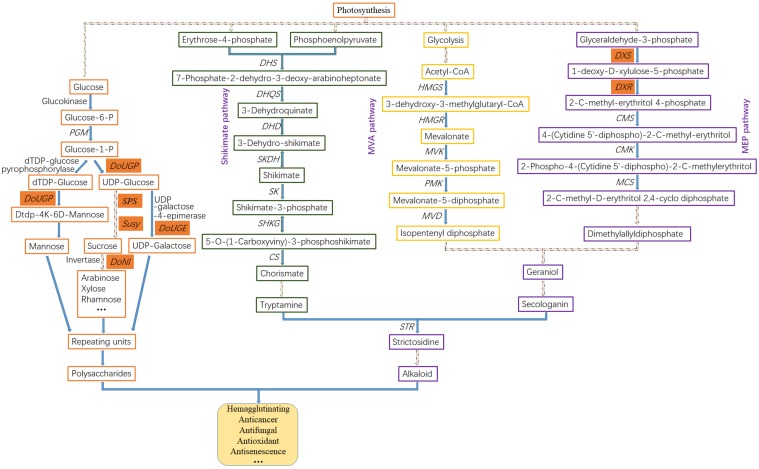
Putative biosynthetic pathways of polysaccharide and alkaloid in *Dendrobium*. In the polysaccharide biosynthesis pathway, monosaccharides (mannose, glucose, galactose, arabinose, xylose, etc.) are produced through hydrolysis or hydrolysis-derivative reactions. These monosaccharides as basic building blocks and repeating units are then used to synthesize polysaccharides. In the alkaloid biosynthesis pathway, the upstream precursors are produced mainly through the Shikimate pathway, and MVA and MEP pathways. Genes in red cells indicate the cloned and functionally studied genes in *Dendrobium*. The dashed lines indicate multiple steps. Enzyme abbreviations are as follows: *PGM*, phosphoglucomutase; *UGP*, UDP-glucose pyrophosphorylase; *SPS*, sucrose-phosphate synthase; *Susy*, sucrose synthase; *DHS*, 3-dexoy-7-phosphoheptulonate synthase; *DHQS*, 3-dehydroquinate synthase; *DHD*, 3-dehydroquinate dehydratase; *SKDH*, shikimate dehydrogenase; *SK*, shikimate kinase; *SHKG*, 3-phosphoshikimate 1-carboxyvinyltransferase; *CS*, chorismate synthase; *HMGS*, hydroxymethylglutaryl-CoA synthase; *HMGR*, 3-hydroxy-3-methylglutaryl CoA reductase; *MVK*, mevalonate kinase; *PMK*, phosphomevalonate kinase; *MVD*, diphosphomevalonate decarboxylase; *DXS*, 1-deoxy-D-xylulose-5-phosphate synthase; *DXR*, 1-deoxy-D-xylulose-5-phosphate reductoisomerase; *CMS*, 2-C-methyl-D-erythritol 4-phosphate cytidylyl transferase; *CMK*, 4-(cytidine 5′-diphospho)-2-C-methyl-D-erythritol kinase; *MCS*, 2-C-methyl-D-erythritol 2,4-cyclodiphosphate synthase; *STR*, strictosidine synthase.

One of the main purposes of transcriptome research in medicinal *Dendrobium* plants is to analyze the biosynthetic pathway and identify the key enzyme genes involved in specialized metabolite production. Several transcriptome studies have focused on the biosynthetic pathway of alkaloids in *Dendrobium* species. For instance, a previous study (Yuan et al., 2018) revealed that the alkaloids in the *Dendrobium* genus are mostly sesquiterpenoid alkaloids or terpenoid indole alkaloids (TIA). Functional analysis based on KEGG terms revealed 25 genes associated with alkaloid backbone construction that belonged to the TIA class by using the Roche 454 GS FLX Titanium platform (Guo et al., 2013; Figure 1). The upstream of the TIA pathway, which can be further divided into terpenoid-forming and indole pathway, is conserved among alkaloid-producing plants and initiated from the shikimate, mevolonate (MVA), or the methylerythritol phosphate (MEP) pathway (Figure 2). In the terpenoid-forming pathway, 10-hydroxylase (G10H) catalyzes geraniol to produce 10-hydroxygenraniol. After a series of enzymatic reactions, 10-hydroxygenraniol is converted to loganin, which is then further catalyzed by secologanin synthase (SCS) to generate secologanin (Wang C.T. et al., 2010). From the indole pathway, tryptamine is synthesized. These two intermediates, secologanin and tryptamine, then combined with each other by strictosidine synthase (STR) to form strictosidine (Zhu et al., 2014), a common precursor for all TIA biosynthesis.

The upstream of alkaloid biosynthesis is mainly through three pathways: the shikimate pathway, the MEP pathway, and the MVA pathway (Figure 2). The shikimate pathway in plants is essential for a variety of second metabolite synthesis (Tzin et al., 2012). A series of key enzymes involved in shikimate pathway have been identified. Among them, 5-enolpyruvylshikimate-3-phosphate synthase (EPSP) is a key enzyme involved in the formation of enolpyruvylshikimate 3-phosphate (Klee et al., 1987). The stem-specific expression of EPSP in *D. huoshanense* enhanced the accumulation of tryptamine, which is a precursor for strictosidine biosynthesis (Yuan et al., 2018). Seventeen unigenes associated with six enzymes were revealed by transcriptome analysis in *D. officinale* and were mapped to the shikimate pathway (Shen et al., 2017), including 3-deoxy-D-arabinoheptulosonate-7-phosphate synthase (DHS), 3-dehydroquinate synthase (DHQS), 3-dehydroquinate acid dehydratase (DHD), shikimate dehydrogenase (SKDH), 5-enolpyruvylshikimate-3-phosphate synthase (SHKG), and farnesyl diphosphate synthase (FPS). In the MEP pathway, 1-deoxy-D-xylulose 5-phosphate synthase (DXS) is the first key enzyme, and 1-deoxy-D-xylulose-5-phosphate reductoisomerase (DXR) is the second and rate-limiting enzyme catalyzing a branched isovaleric precursor to form a straight chain pentose sugar (Ramak et al., 2013). Overexpression of *DXS* in *Spike lavender* or *DXR* in *Salvia miltiorrhiza* resulted in a significant increase in terpenoid accumulation (Munoz-Bertomeu et al., 2006). In the MVA pathway, 3-hydroxy-3-methyl-glutaryl-coenzyme A reductase (HMGR) is one of the key enzymes involved in terpenoid biosynthesis. It is capable of catalyzing 3-hydroxy-3-methylglutaryl coenzyme A (HMG-CoA) to form MVA. Overexpression of *HMGR1* in ginseng enhanced the steroid and triterpene production (Kim et al., 2014), suggesting the promoting role of *HMGR1* in ginsenoside biosynthesis.

**FIGURE 3:**
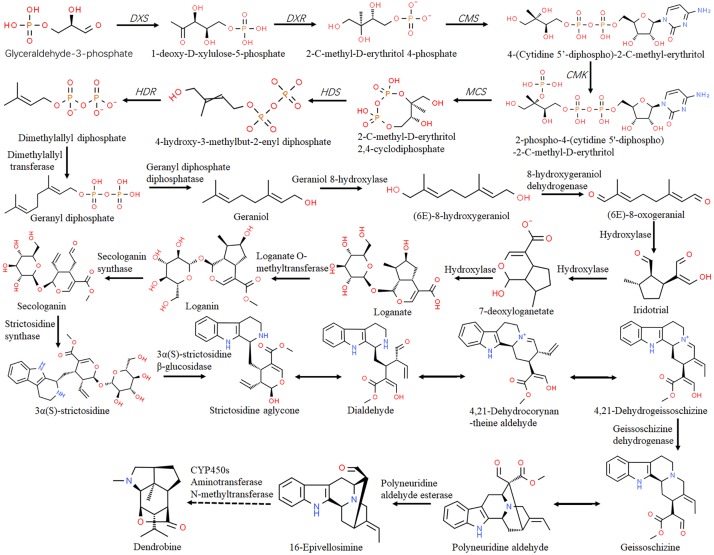
Biosynthesis of dendrobine in *Dendrobium* through the MEP pathway. The putative pathway was adapted from Li Q. et al. (2017) and Zheng et al. (2018). The enzymes or enzyme encoding genes were indicated alongside the arrows. The dashed lines indicate multiple steps.

Previous reports revealed 34 alkaloids isolated from 14 *Dendrobium* species, 21 of which were dendrobine alkaloids with a sesquiterpene skeleton structure (Xu et al., 2017). A pharmacology study demonstrated that dendrobine restrains the growth of *A549* lung cancer cells and acts as a promising agent for treating virus infection (Li R. et al., 2017). The upstream biosynthetic pathway of dendrobine is composed of the MVA and MEP pathway, which are conserved to provide basic skeleton for terpenoid alkaloids (Figure 2). Both pathways can produce isopentenyl diphosphate (IPP), which is the precursor of synthetic terpenes that can be exchanged on the plasma membrane. Key enzymes involved in the MEP pathway (Figure 3) including DXS and DXR, as well as enzymes functioning in the MVA pathway such as hydroxymethylglutaryl-CoA synthase (HMGS) and HMGR have been annotated in *D. officinale* (Fan et al., 2016; Chen et al., 2019). In *D. nobile*, large-scale transcriptome analysis has been generated in response to *MF23* infection with increased dendrobine production (Li R. et al., 2017). From the datasets, 16 genes encoding acetyl CoA acetyltransferase (AACT), phosphomevalonate kinase (PMK), diphosphomevalonate decarboxylase (MVD), and terpene synthase 21 (TPS21), which are members related to the biosynthesis of the backbone of sesquiterpene alkaloid dendrobine, have been identified, suggesting the dominant role of the MVA pathway in this process. In addition, 11 genes encoding nine enzymes were mapped onto the MEP pathway (Figure 3), which serves as a supplemental provider of isoprene units for dendrobine biosynthesis.

### Post-modifications

Following the generation of strictosidine, the alkaloid biosynthesis in *Dendrobium* is characterized mainly by a set of post modifications (PTM), such as cytochrome P450s (CYP450s)-mediated oxidation and hydroxylation reactions (Guo et al., 2013). As a complex superfamily of monooxygenase, CYP450s play key roles in specialized metabolite biosynthesis, and some of them have been isolated and characterized (Seki et al., 2008). For instance, annotation of the 454 EST pool of *D. officinale* against the SwissProt database revealed 93 *CYP450*s transcripts belonging to 17 families (Guo et al., 2013). Among them, transcripts of the *CYP71* (9.7%) were likely to be involved in hydroxylation steps of alkaloid biosynthesis. In *D. huoshanense*, 229 unigenes were identified as putative *CYP450*s, most of which were also the *CYP71* family members (7.8%), followed by *CYP3A* and *CYP4* family members (Yuan et al., 2018). Moreover, several post-modification enzymes involved in the biogenic pathway of dendrobine, such as CYP450s, aminotransferase, and methyltransferase (Figure 3), have been identified in *D. nobile* (Li Q. et al., 2017) and *D. officinale* (Chen et al., 2019). Among them, *CYP1D10*, *METTL23*, *ATX4*, and *BCAT2* were significantly upregulated in response to *MF23* infection (Li Q. et al., 2017), suggesting their positive roles in promoting dendrobine biosynthesis.

Transcription factor (TF) also plays a vital role in coordinating the expression of alkaloid biosynthesis genes, thus affecting the constituents’ composition in higher plants (Table 3). For example, the AP2/ERF family members ORCA2 and ORCA3 were capable of regulating a subset of alkaloid biosynthesis genes (Van Der Fits and Memelink, 2000). Additionally, a large number of TFs, including C3H, bHLH, bZIP, MYB, and WRKY (Yuan et al., 2018), have been previously reported to play central roles in the regulation of alkaloid biosynthesis in *Dendrobium*.

**TABLE 3:** Transcription factor families identified in the medicinal *Dendrobium* transcriptome datasets.

No. of unique transcripts	Guo et al., 2013	Zhang J. et al., 2016	Yuan et al., 2018	Chen et al., 2019
bHLH	72	75	145	93
bZIP	49	23	227	23
WRKY	44	60	71	71
MYB	36	82	64	95
NAC	36	24	69	61
GRAS	24	4	37	15
MADS	13	30	28	25
TCP	13	6	24	25

## *Dendrobium* Polysaccharide Biosynthesis

### *Dendrobium* Polysaccharide

Many *Dendrobium* plants are precious medicinal herbs partially because of their abundant polysaccharides found in stems, leaves, and flowers. In recent years, many soluble polysaccharides were extracted from various *Dendrobium* species, and the structure, composition, and bioactivity were determined (He et al., 2015). The major polysaccharides in *Dendrobium* stems were non-starch mannan polysaccharides, to a lesser extent glucose, and a small amount of galactose (Ng et al., 2012). Semithin and ultrathin sections demonstrated that polysaccharides that formed granules were stored in the plastids of stems (He et al., 2017a), similar to starch grains.

### *Dendrobium* Polysaccharide Biosynthesis

Polysaccharides are one of the major medicinal components in *Dendrobium* plants with antitumor, antioxidant, antiaging, antibacterial, antiviral, antiradiation, and anticoagulant activities (Liu et al., 2011; Wang et al., 2014). Thus far, a large number of carbohydrate-related genes (Li Q. et al., 2017) encoding glycosyltransferase (GT), glucosyltransferase, and mannosyltransferase, most of which were expressed more highly in stems than in leaves and roots, have been identified and might play vital roles in polysaccharide synthesis (Figure 2). Enzymes directly involved in polysaccharide metabolism, such as GDP-mannose pyrophosphorylase (GMP) in *Dendrobium*, have also been characterized (He et al., 2017c). Moreover, it has been confirmed that sucrose synthase (Susy) was positively correlated with the polysaccharide content in *D. officinale* and *D. huoshanense* and that two unique gene families, the *galacturonosyltransferase* and β*-galactosidase* gene families, were related to the richness of polysaccharides in *D. officinale* (Yan et al., 2015). GT is a group of carbohydrate-active enzymes that catalyzes the glycosidic bond formation in glycan and glycoside biosynthesis by transfer of sugar moieties from active donors to acceptor molecules (Shao et al., 2005; Lao et al., 2014). A recent transcriptome analysis in *D. officinale* (Shen et al., 2017) identified 280 *GT*s, including genes encoding glucosyltransferase (236), fucosyltransferase (11), mannosyltransferase (16), and xylosyltransferase (17) using BLASTX methods.

Mannan polysaccharides are the major component of polysaccharides from most of the *Dendrobium* species, accounting for as much as 58.3% of the dry weight of the crude polysaccharide fraction in *D. officinale* (Xing et al., 2015), and also promising bioactive ingredients for use in drugs. It has beneficial effects on human health with increased cytokine production and antioxidant and anticancer activities (Xing et al., 2015). Mannan polysaccharides can be further classified into four subfamilies (Buckeridge, 2010): pure mannan, glucomannan (GM), galactomannan (GGM), and galactoglucomannan. The biosynthesis of mannan polysaccharides is mediated by mannan synthases using GDP-D-mannose or GDP-D-glucose as substrates (Hassid, 1969). Moreover, the *cellulose synthase A* (*CesA*) superfamily genes have also been demonstrated to be involved in the biosynthesis of mannan polysaccharides (Lerouxel et al., 2006). The CesA superfamily can be subdivided into one cellulose synthase (CesA) family and nine cellulose synthase-like (Csl) families, CslA to CslJ (Suzuki et al., 2006). In *D. officinale*, eight *CslA* genes (*DoCslA1* to *DoCslA8*) were identified and analyzed to provide genetic evidences for their roles in mannan polysaccharide biosynthesis (He et al., 2017a). Overexpression of *DoCslA6* increased mannose content in *Arabidopsis thaliana* (He et al., 2017a). In addition, GDP-mannose transporter (GMT), which translocates GDP-mannose into the Golgi lumen, is indispensable for mannan polysaccharide biosynthesis. Three *GMT* genes, *DoGMT1* to *DoGMT3* (Yu et al., 2018), have been identified in *D. officinale* with the highest transcript levels in stems.

### Polysaccharide Synthesis and Sucrose Metabolism

Polysaccharide synthesis and sucrose metabolism are closely linked because many monosaccharides, the basic building blocks for polysaccharide synthesis, are produced from sucrose hydrolysis or hydrolysis derivatives. In general, sucrose metabolism involves two distinct processes, sucrose synthesis and sucrose breakdown, which are mainly catalyzed by sucrose phosphate synthase (SPS) and Susy, respectively (Huber and Huber, 1996). A previous study revealed that the levels of polysaccharides in *D. officinale* were closely related to the concentrations of the reduced sugar and soluble sugar, which were directly affected by sucrose invertase and SPS activities (Yan et al., 2015). Yan Liang and colleagues (Yan et al., 2015) analyzed the genome sequence of *D. officinale* and identified 10 *SPS* and 15 *Susy* genes, which have undergone marked expansion through tandem duplication. Likewise, in *D. huoshanense*, 13 *SPS* and 18 *Susy* genes have been isolated (Yuan et al., 2018). Alkaline/neutral invertase (NI) is responsible for sucrose hydrolysis to produce glucose or fructose in the cytoplasm. An *NI* gene, *DoNI*, was cloned in *D. officinale* by the rapid amplification of cDNA ends (RASE) method. The expression of DoNI was associated with its activities in different tissues and, more importantly, the polysaccharide accumulation (Gao et al., 2016). Uridine diphosphate glucose (UDPG) is an important direct or indirect glycosyl donor for synthesis of polysaccharides. The UDPG pyrophosphorylase (UDPase) is a key enzyme for reversibly catalyzing UDPG into glucose-1-phosphate (Glc-P), which is then utilized in synthesis of polysaccharides by GTs. A novel UGPase gene, *DoUGP*, was identified from *D. officinale* (Wan et al., 2017). It was highly expressed in stems in comparison to other organs and positively correlated with the highest polysaccharide content there (Wan et al., 2017). Sucrose feeding significantly increased *DoUGP* expression and enhanced polysaccharide production accordingly in both protocorm of *D. officinale* and protocorm-like bodies of *D. huoshanense* in suspension cultures (Wan et al., 2017). Thus, *DoUGP* is probably involved in polysaccharide synthesis and might serve as a potential target for quality breeding of *Dendrobium* orchids.

## Biosynthesis of Other Compounds in *Dendrobium*

### Tropine Biosynthesis-Related Genes

Tropine is an alkaloid derived from tropinone, which can be reduced by tropinone reductase (TRs) using the NADPH as coenzyme (Nakajima et al., 1998). TRs can be further divided into two subgroups, TRI and TRII, based on the stereospecificity of reduction product. TRI is responsible for tropine production, whereas TRII is mainly involved in the generation of pseudotropine (Nakajima et al., 1998). Most of *TR* homologous genes found in other plant species do not have tropinone reduction activity, except for plants belonging to or closely related to the *Solanaceae* family (Drager, 2006) and *CoTR* from *Cochlearia officinalis* (Brock et al., 2008). Recently, *DnTR1* and *DnTR2* that encode peptides with similarity to known *TR*s were cloned from *D. nobile*. Catalytic activity assay revealed that both DnTR1 and DnTR2 were able to reduce 3-quinuclidinone hydrochloride and 4-methylcyclohexanone using NADPH as coenzyme (Chen et al., 2013). Moreover, DnTR1 could reduce tropinone, whereas DnTR2 couldn’t (Cheng et al., 2013), implying their tremendous variation in substrate specificity.

### Flavonoid Biosynthesis-Related Genes

Flavonoids are the second most common compounds in *D. officinale*, exhibiting diverse medicinal functions including antioxidant and protective effects on cell toxicity and treatment of various degenerative and age-related diseases (Prochazkova et al., 2011; Wang et al., 2017). Most flavonoids in *Dendrobium* are C-glycosides with basic skeletons including vitexin, quercetin, luteolin, apigenin, etc. (Lei et al., 2018). The biosynthesis of most flavonoids begins in the phenylpropanoid pathway using malonyl-CoA and p-coumaroyl-CoA as precursors (Liu et al., 2013). The whole processes are regulated by many key enzymes, transcription factors, UDP-GT, and CYP450s (Liu et al., 2013). Transcriptome analysis (Lei et al., 2018) revealed that 31 unigenes encoding 14 enzymes were involved in the biosynthesis of flavonoids in *D. catenatum*. Synthesis of the three basic flavonoid glycoside skeletons are regulated by flavonol synthase (FLS), CYP75A, and flavonoid 3′-monooxygenase. Specifically, FLS is involved in the transformation of dihydroquercetin to quercetin, and dihydrokaempferol to kaempferol, which can be subsequently catalyzed into quercetin by CYP75A and flavonoid 3′-monooxygenase (Lei et al., 2018), two enzymes also regulating the synthesis of luteolin from apigenin.

## Strategies for Enhancing *Dendrobium* Bioactive Compounds Production

Elicitation and precursor feeding are two major effective methods for increasing the accumulation of specialized metabolites (Skrzypczak-Pietraszek et al., 2014). Mycorrhizal fungi isolated from the roots of wild *D. officinale* and *D. nobile* can serve as an elicitor for seed germination and specialized metabolite production (Xu et al., 2014), by offering nutrients such as glucose directly to their hosts, or through secreting certain types of phytohormones supplied to the hosts (Zhang, 1999). For instance, inoculation of *D. nobile* with *MF23*, a mycorrhizal fungus previously isolated from the roots of *D. officinale*, significantly increased total alkaloid content (18.3%) by forming peloton to supply nutrients for their hosts (Zhang et al., 2012). Similarly, *Ceratocystis fimbriata* infection significantly increased alkaloid accumulation in mango (Araujo et al., 2016).

Apart from the biotic factors, some abiotic stresses induced by unfavorable environments (drought, salt, etc.) and stress hormones (methyl jasmonate, MeJA) can also promote specialized metabolite accumulation. For example, the alkaloid biosynthesis was markedly increased in *Catharanthus roseus* and *Motherwort* by binary stress and drought stress, respectively (Wei et al., 2013; Zhu et al., 2015). The phytohormone MeJA has been identified as a signaling molecule that switches on gene expression and enhances the biosynthesis of various bioactive compounds, particularly alkaloids and polysaccharides in medicinal plants including *Dendrobium* species (Zhan et al., 2018; Zhang et al., 2018). Exogenous feeding of MeJA enhanced the catalytic efficiency and the expression of strictosidine synthase (STR), which plays a vital role in alkaloid biosynthesis (Paul et al., 2017). Additionally, precursor feeding has also been performed to increase the alkaloid/polysaccharide production, such as tryptamine application to enhance reserpine synthesis in *Rauvolfia serpentina*; tryptophan, tryptamine, secologanin, and loganin feeding to promote the accumulation of ajmalicine, vindoline, and catharanthine in *C. roseus* (Panwar and Guru, 2015). Likewise, sucrose feeding (Wan et al., 2017) upregulated *DoUGP* transcription, and correspondingly increased polysaccharides content in *D. officinale*.

## Perspectives

**FIGURE 4:**
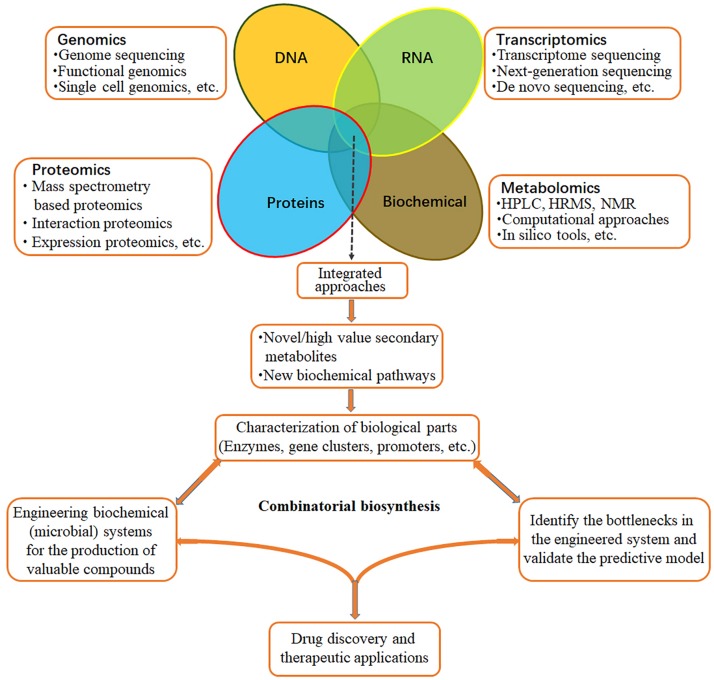
Integrated approaches for production of bioactive medicinal ingredients. Combining multiple “omics” datasets will facilitate the discovery of novel bioactive specialized metabolites and characterization of the related biosynthesis pathways in *Dendrobium* species. Based on the obtained knowledge, novel combinatorial biosynthesis systems can be engineered for the production of new and interesting specialized metabolites.

*Dendrobium* genus in *Orchidaceae* is well known worldwide for its high economic and medicinal values. Recently, transcriptomes of *Dendrobium* have been sequenced for validation of genes involved in specialized metabolite biosynthesis. The resultant datasets will contribute to further research on metabolic pathways, molecular genetic breeding, genetic engineering, excavation, and protection of genetic resources of medicinal *Dendrobium* plants. Along with the progress of sequencing technology, novel strategies of targeted isolation, purification, nuclear magnetic resonance (NMR) identification, proteomics, and metabolomics will facilitate the full exploration of the molecular mechanism of bioactive ingredient biosynthesis regulation (Figure 4). Meanwhile, a great many of the genes associated with post-modifications (PTMs) have been identified. They are complex but evolutionarily conserved biochemical modifications consisting of hundreds of directly or indirectly intertwined reactions in various eukaryotic and prokaryotic cells. Although transcriptome analysis revealed that the expression levels of some genes encoding post-modification enzymes, such as CYP450s, aminotransferases, and methyltransferases were upregulated during bioactive compounds production, future research regarding the downstream of PTM processes should be performed. Moreover, abiotic (cold acclimation, light intensity, water, salt stress, etc.) and biotic (fungus infection) stresses and precursor feeding can significantly influence the expression of numerous genes involved in metabolome and the accumulation of metabolites in *Dendrobium*. Therefore, it is feasible to improve active ingredients production by the combinations of stress stimulation and precursor feeding. Even though the engineering of specialized metabolite production is quite challenging due to incomplete and uncertain information, combinatorial biosynthesis (Figure 4) that reconstitutes genes from plant metabolomic pathways in microorganisms or other plant species for the targeted ingredients biosynthesis, or combines genes from different microorganisms for the production of new and interesting plant specialized metabolites is of great potential for further investigation.

## Author Contributions

ZW and MW conceived the project. ZW drafted the manuscript. ZW, MZ, and HC evaluated and interpreted the data. JL and MW revised the manuscript. All authors read and approved the final manuscript.

## Conflict of Interest

The authors declare that the research was conducted in the absence of any commercial or financial relationships that could be construed as a potential conflict of interest.
